# Female reproductive functions of the neuropeptide PACAP

**DOI:** 10.3389/fendo.2022.982551

**Published:** 2022-09-20

**Authors:** Miklos Koppan, Zsuzsanna Nagy, Inez Bosnyak, Dora Reglodi

**Affiliations:** ^1^ Maternity Clinic, Budapest, Hungary; ^2^ Department of Physiology, University of Pecs Medical School, Pécs, Hungary; ^3^ Department of Anatomy, ELKH-PTE PACAP Research Group and Szentagothai Research Center, University of Pecs Medical School, Pécs, Hungary

**Keywords:** PACAP, GnRH, LH, FSH, ovary, placenta

## Abstract

Pituitary adenylate cyclase activating polypeptide (PACAP) is a neuropeptide originally isolated as a hypothalamic peptide. It has a widespread distribution in the body and has a diverse spectrum of actions. Among other processes, PACAP has been shown to be involved in reproduction. In this review we summarize findings related to the entire spectrum of female reproduction. PACAP is a regulatory factor in gonadal hormone production, influences follicular development and plays a role in fertilization and embryonic/placental development. Furthermore, PACAP is involved in hormonal changes during and after birth and affects maternal behavior. Although most data come from cell cultures and animal experiments, increasing number of evidence suggests that similar effects of PACAP can be found in humans. Among other instances, PACAP levels show changes in the serum during pregnancy and birth. PACAP is also present in the human follicular and amniotic fluids and in the milk. Levels of PACAP in follicular fluid correlate with the number of retrieved oocytes in hyperstimulated women. Human milk contains very high levels of PACAP compared to plasma levels, with colostrum showing the highest concentration, remaining steady thereafter for the first 7 months of lactation. All these data imply that PACAP has important functions in reproduction both under physiological and pathological conditions.

## Introduction

PACAP was discovered in the laboratory of professor Arimura in 1989 (1). The discovery was based on finding a novel hypothalamic peptide that stimulated anterior pituitary cells in addition to the already known releasing hormones. This led to the isolation of a peptide composed of 38 amino acid residues, named PACAP38, from ovine hypothalamic extracts. This was followed by the isolation of a shorter form with 27 amino acids, named PACAP27 (2).The name PACAP comes from the abbreviation of pituitary adenylate cyclase activating polypeptide, referring to the first described action, in which it stimulates adenylate cyclase activity, and thus, cAMP in the pituitary gland. Both peptides show structural homology to the vasoactive intestinal peptide (VIP) and they belong to the VIP/secretin/glucagon peptide family.

PACAP acts through G protein-coupled receptors, namely the specific PAC1 and VPAC1 and VPAC2, which also bind VIP with equal affinity (2). PACAP activates mainly the adenylate cyclase/cAMP pathways, and through this, the activation of its receptors lead to activation of protein kinase A (PKA) and downstream pathways (3). It also activates several other pathways (4) and transactivates tyrosine kinase receptors (5). Moreover, PACAP38 (but not PACAP27) can enter through the cell membrane without receptorial mechanism, but the intracellular signaling activated this way has not been elucidated yet (6). The specific PAC1 receptor has several splice variants, inducing different signaling pathways, and thus, leading to different, sometimes opposing effects (2, 7).

PACAP and its receptors have widespread occurrence and thus, PACAP can exert variable biological actions. In the nervous system, it acts as a neurohormone and neuromodulator, and several different effects have been described. Among others, it plays a role in neuronal development like patterning of the neural tube, proliferation and migration of cortical and cerebellar neurons, axonal growth and glial cell maturation (8). These effects can also be observed in the mature nervous system in case of injuries, when PACAP can exert neuroprotective effects (9–12). Several other neuronal processes are influenced by PACAP: it has been shown to play a role in stress and anxiety responses (13), in diminishing the negative consequences of aversive events (14), it influences central energy homeostasis (15, 16), thermoregulation (17) and memory (18). In the periphery (19, 20), several actions have been described regarding the cardiovascular system, where the peptide influences cardiac neuronal excitability and heart muscle contractility (21). In the gastrointestinal and respiratory tract PACAP plays a role in neuroendocrine secretion, smooth muscle contractility and blood supply (22, 23). Endocrine glands show high levels of expression of PACAP and the peptide is involved in secretion of several hormones (19–23).

Regarding reproductive functions, several lines of evidence show that the peptide centrally regulates gonadal hormones as well as acts in the periphery, the ovary, the placenta, the mammary gland and the uterus. Male reproductive functions are also known to be influenced by PACAP at both central and peripheral levels. At central level, PACAP influences hypothalamic and hypophyseal gonadal hormone secretion, while in the periphery, PACAP regulates spermatogenesis at various stages (24–26), influences sperm cell motility (27), and modulates Leydig and Sertoli cell functions (28–30). The reproductive functions of PACAP seem to be evolutionarily conserved, as it has also been revealed in several non-mammalian species (31). In seasonal animals, PACAP expression and effects are season-dependent (32), while in non-seasonal breeding species, the PACAP system shows alterations throughout the life-span, before and after puberty, during the hormonal cycle and during pregnancy (33). The aim of the present mini-review is to summarize findings regarding the effects of PACAP in the female reproductive system.

## PACAP in the central regulation of female reproductive functions

Soon after the discovery of PACAP it became evident that the neuropeptide plays a role in modulating the secretion of releasing hormones, such as the main hypothalamic hormone playing a role in gonadal regulation, gonadotropin-releasing hormone (GnRH) and pituitary hormones, including follicle-stimulating hormone (FSH) and luteinizing hormone (LH) influencing peripheral reproductive functions. Early studies revealed that PACAP occurs at highest concentrations in the hypothalamus, although several other brain areas express significant amount of the peptide as well (2). Hypothalamic neuronal endings release PACAP in the median eminence where the primary capillary plexus of the hypophyseal portal system is found. The concentration of PACAP in the hypophyseal portal venous blood has been shown to be higher than in the periphery, proving the release and transport of the peptide to the adenohypophysis (34). PACAP is thus carried *via* the portal vessels to the anterior pituitary where it acts, among others, on the gonadotroph cells. Strong PACAP immunoreactivity was found in several hypothalamic nuclei, such as arcuate, dorsomedial, ventromedial, paraventricular nuclei, lateral and preoptic hypothalamic areas. mRNA has also been shown in the perikarya of some of these nuclei. Regarding PACAP binding sites, receptors PAC1 and VPAC1/2 are present in many brain areas. In the hypothalamus, receptors have been identified in the arcuate, dorsomedial, ventromedial, paraventricular, supraoptic, preoptic and suprachiasmatic nuclei, in the lateral hypothalamic area and in the mamillary bodies. These data mostly come from rat experiments, but subsequent studies have also mapped PACAP and its receptors in several other species, including the human brain (35–37). Distribution of PACAP in the human hypothalamic nuclei closely resembles that described in rodents (35), underlining the translational value of the rodent studies. Various hypothalamic functions are influenced by PACAP. For example, PACAP is involved in the hypothalamic regulation of body temperature (38, 39), food and water intake (40–43), energy homeostasis (16, 44) and in the circadian rhythmic activity of the suprachiasmatic nucleus (45). All these hypothalamic actions are in a complex interplay with the regulatory mechanisms of reproductive functions.

The hypothalamic nuclei playing a role in the hypothalamo-hypophyseal hormonal system can be divided into magnocellular and parvocellular nuclei. Magnocellular nuclei are the supraoptic and paraventricular nuclei that produce vasopressin and oxytocin, both of which are transported by axonal transport *via* the hypothalamohypophyseal tract to the posterior lobe of the pituitary gland, where they are released into the bloodstream. High expression of both PACAP and its receptors are found in these nuclei. Intracerebral injection of PACAP increases activity of these neurons and plasma vasopressin levels (46–48). PACAP increases oxytocin and vasopressin release in the posterior lobe of the hypophysis (49). Parvocellular nuclei of the hypothalamus are mainly involved in the production of releasing hormones that influence the production of anterior pituitary trophic hormones, such as FSH, LH, thyroid-stimulating hormone (TSH), adrenocorticotropic hormone (ACTH), growth hormone (GH) and prolactin (PRL). The main parvocellular nuclei are the ventromedial, dorsomedial, preoptic, arcuate nuclei and the parvocellular part of the paraventricular nucleus. PACAP has been proven to act as a modulator and transmitter in the regulation of hypophysiotropic hormones in the parvocellular system (2). Several lines of evidence prove that PACAP is involved in the GnRH-gonadotropin axis (50, 51). PACAP leads to an increase in the gene expression of GnRH, somatostatin and corticotrope-releasing hormone (CRH), while the injection of the PACAP antagonist PACAP6-38 inhibits this increase.

In the adenohypophysis, PACAP receptors are found in all endocrine cells and also in folliculostellate cells (2). Moore et al. (52) investigated the expression of PACAP mRNA during the estrus cycle. They found that PACAP mRNA expression in the paraventricular nucleus and pituitary shows significant changes during the estrous cycle, with the greatest alterations on the day of proestrus. PACAP mRNA in the paraventricular nucleus decreases on the morning of diestrus, while increases 3 h prior to the gonadotropin surge and then declines in proestrus. A moderate decline at the time of the gonadotropin surge on the afternoon of proestrus and an increase later in the evening was observed in the pituitary. Expression of the follistatin mRNA increased following the rise in pituitary PACAP mRNA, after the secondary surge in FSH beta (Fshb) gene expression. They concluded that PACAP is involved in events before and after the gonadotropin surge, possibly through increased sensitivity to GnRH and suppression of Fshb subunit expression, similarly to *in vitro* observations (52). Others have also confirmed the rise of PACAP in the anterior pituitary during proestrus (53–55).

Early studies have shown that PACAP stimulates release of GH, ACTH, LH, FSH and PRL (2). PACAP can alone stimulate LH and FSH but also acts synergistically with GnRH (2). PACAP also stimulates GnRH receptor gene promoter activity, while GnRH stimulates PACAP gene expression, highlighting the complex relationship between PACAP and GnRH systems (2). This complexity is further deepened by the somewhat contradictory results regarding the relationship between PACAP, GnRH and the gonadotrophs. Although some studies found no effect of intravenous PACAP administration on LH levels (56, 57), the same authors described inhibition of the LH surge when PACAP was administered intracerebroventricularly. Interestingly, PACAP27 and 38 had opposing effects: while PACAP38 inhibited LH surge, PACAP27 elevated LH plasma levels. Also, PACAP38 inhibited ovulation when given intracerebroventricularly or intranasally, while PACAP27 had no effect on it (57, 58). Others also found inhibitory action of PACAP on LH release (59). Similarly, injection into another area, the medial basal hypothalamus, led to decreases in LH secretion, LH pulse frequency and ovulation (59). Contradictory results show that the relationship between PACAP and the gonadotropin axis is very complex, as other studies have found stimulatory action on LH release (60–62). Most probably the opposite findings can be explained by the different experimental setups, as it was shown that the action of PACAP depends on the age of the animal, time of day, gender, the day of the estrous cycle, GnRH pulse frequency, using PACAP27 versus 38, and there are differences also between *in vitro* and *in vivo* findings (63). This complex system and the effects of PACAP were thoroughly and critically analyzed in the review by Koves et al. (51).

The first studies on PACAP and the onset of puberty showed that neonatally administered subcutaneous PACAP delayed puberty and a lower number of eggs were released at ovulation, accompanied by lower pituitary LH content (64). Another study revealed that disruption of PAC1 receptor synthesis delayed puberty and decreased GnRH receptor and LH in the pituitary (65). A further insight into the complexity of the interaction between PACAP and gonadotropin system comes from studies investigating other influencing factors, such as other releasing hormones, interleukins, estradiol and progesterone, and several neuropeptides (51). Recent results indicate that PACAP acts also *via* kisspeptin neurons on GnRH secretion. While PACAP can affect GnRH neurons in the hypothalamus directly or indirectly through CRH, it can also influence kisspeptin neurons which create the pulse generator (51). The relationship between kisspeptin and PACAP was suggested in studies by Mijjiddorj et al. (66). These investigations have shown that PACAP and kisspeptin synergistically increase gonadotropin subunit expression, Cre promoter expression, prolactin-promoter activity and kisspeptin increases the expression of PAC1 receptor (66, 67). Tumurbaatar and colleagues have confirmed the relationship between kisspeptin neurons and PACAP, as they showed a stimulation of the gene encoding kisspeptin by PACAP in hypothalamic cells derived from the kisspeptin-expressing periventricular and arcuate nuclei (68).

PACAP or PAC1 receptor knockout animals have high mortality and lower reproduction rate (69–71). PACAP KO mice have numerous abnormalities and pathological symptoms with several biochemical and developmental alterations (71–76). No exact explanation for the lower fertility is known, but several factors seem to play a role, including hormonal differences. Although most authors working with knockout animals have described decreased fertility, differences can be found in the background. While some investigators found no difference in the onset of puberty and estrous cycle, others have found disturbed estrous cycle (70, 77). Isaac and Sherwood (78) described lower implantation rate associated with reduced prolactin and progesterone levels. Shintani et al. (70) reported reduced mating and maternal behaviour. Immune-checkpoint molecules were investigated in decidual and peripheral immune cells in the periphery and in the decidua of pregnant KO mice. The only noteworthy finding was the recruitment of galectin-9 Th cells to the decidua promoting local immune homeostasis in PACAP KO mice, but this difference alone is not significant enough to explain the background of the reduced fertility, but point to a role of PACAP in the immune regulation of pregnancy (79). A pioneer study by Ross et al. (80) provided more insight into the relationship between neurons expressing PACAP, kisspeptin or leptin, and thus, providing a possible explanation for the altered estrous cycle seen in some studies. They showed that the main site of leptin receptor and PACAP co-expression is the ventral premamillary nucleus of the hypothalamus. A targeted deletion of PACAP from this nucleus led to delayed onset of puberty, measured by delayed vaginal opening and first estrous cycle. These mice had also dysregulated estrous cycle later and had impaired reproductive functions, as pregnant mice had fewer pups per litter. These were accompanied by blunted LH surge and a smaller number of follicles maturing per cycle. As the PACAP/leptin neurons project to kisspeptin neurons, a new role for PACAP-expressing neurons has been suggested based on these observations: PACAP expressing neurons in the ventral premamillary nucleus play a role in the relay of nutritional status to regulate GnRH release by modulating kisspeptin neurons (80). Our preliminary results also confirm this relationship between PACAP and kisspeptin expression and disturbed estrous cycle in PACAP deficient animals (77). Altogether, studies on PACAP and the hypothalamo-hypophyseal system clearly show that PACAP plays a role in the central reproductive functions, but more studies are needed to resolve the controversies in the hormonal regulation. Furthermore, the lack of human data in this regard makes the translational value of these studies questionable, as the reproductive functions are well-known to be highly species-specific.

## PACAP in the gonads

It is well known that interactions between peptide and steroid hormone-signaling cascades influence the growth of follicles, ovulation, and luteinization in the ovary. Following the gonadotropin-independent follicular development, a cohort of hormone sensitive follicles are selected that rapidly grow into immature and mature tertiary follicles. LH surge induces ovulation and the formation of the corpus luteum from the remaining granulosa and theca cells of the follicles. Follicles produce estrogen, while corpus luteum is responsible for both estrogen and progesterone production. Although FSH and LH play a fundamental regulatory role in follicular maturation, synthesis of steroids, and ovulation, several peptidergic and non-peptidergic signaling pathways may alter their actions (81–85). Influence of PACAP in gonadal functions is further supported by research data showing that PACAP reduces follicular apoptosis in the ovary (86). Follicular development might also correlate with concentrations of PACAP in granulosa cells. In the rat, PACAP expression in the granulosa cells of large mature follicles prior to ovulation is stage-specific, whereas weaker expression could be detected in immature antral and pre-antral follicles (87–89). Both PACAP and PAC1 receptors are found in the rat corpus luteum (90). Moreover, PACAP might also be involved in the regulation of primordial germ cell proliferation (91), as well as cyclic recruitment of immature follicles (89), follicular apoptosis (86, 92), and ovarian hormone and enzyme production in humans, rats, and cows (93–96). These effects have been reviewed by our research group (97) and by Canipari and colleagues (98). A recent study has suggested a novel link between kisspeptin and PACAP at the ovarian level: suppressed PACAP expression after ablation of kisspeptin signaling in oocytes may be an additional factor in the ovulatory failure in mice (99). These studies clearly indicate that PACAP plays a role in follicular development, both through hormonal interactions and locally, influencing oogenesis.

## PACAP in the uterus and placenta

Isaac and Sherwood reported a lower rate of reproduction in PACAP deficient mice, mainly due to insufficient implantation (78). Although the uterine and placental functions of the neuropeptide are somewhat neglected in the literature, findings of the above study might indicate a placental role of endogenous PACAP. Expression of PACAP27 and PACAP38 in human placentas and uterus was first confirmed by radioimmunoassay and immunocytochemistry (100). The uterus consists of a body and cervix, with an isthmus at the border of the two parts. The uterine wall has three layers: endometrium (a columnar epithelial layer), myometrium (a thick smooth muscle layer) and perimetrium (a part of the peritoneum with a thin squamous cell layer) from inside to outside. After implantation, the placenta is formed, consisting of a maternal and a fetal part. Maternal part is the decidua basalis, made up of the pregnant endometrium facing the embryo, while the fetal part consists of the chorion frondosum, which has cyto-and syncytiotrophoblast cells and extraembryonic mesoderm. In the human placenta, PACAP38 concentrations were higher than PACAP27 levels, uterus and placenta had similar levels of immunoreactivity, but the umbilical cord showed weaker intensity (100). Uterine isthmus and myometrium showed stronger immunoreactivity than pregnant uterus, but no immunoreactive nerve fibers could be detected in the placenta or umbilical cord. Radioimmunoassay studies have revealed similar levels of PACAPs in different parts of the human placenta (central/peripheral maternal, central/peripheral fetal). PACAP38-like immunoreactivity was stronger in both maternal and fetal sides in full-term placenta compared to younger samples, while PACAP27-like immunoreactivity increased only on the maternal side (101). Similar to the above data, Scaldaferri and colleagues observed PACAP and PAC1 receptor in both rat and full-term human placentas using Northern blot analysis, polymerase chain reaction (PCR) and immunohistochemistry (102). In human placentas, a marked difference was observed in the immunohistochemical staining characteristics of different parts of the placenta, showing strong staining in stromal cells around blood vessels and weaker signal in vessel walls in stem villi. In terminal villi, stromal cell PACAP38 immunoreactivity was obvious. In stem villi, the stromal immunoreactivity showed a spatial distribution pattern with immunoreactivity only in the periphery, while terminal villi had dispersed positivity in the entire stroma. RT-PCR studies have revealed expression of different isoforms of the PAC1 receptor in rat and human placentas. In the rat placenta, 3 isoforms were described: the short, hip or hop variant and the hip-hop variant. In contrast, in the human placenta only expression of the SV2 form was detected, that is homologous to the rat hop form. PACAP27, PACAP38 had almost equipotent binding to these receptors, while VIP had weaker binding affinity (102).

PAC1 receptor mRNA has been recently demonstrated in the uterus of healthy pigs, and the abundance of PAC1 receptor protein was reduced in inflammatory conditions (103). Endometrial inflammation also leads to changes in PACAP expression of the dorsal root ganglia supplying the porcine uterus (104). PACAP is expressed in the cervix, lumbosacral dorsal root ganglion and spinal cord supplying the uterus, showing time-dependent changes during pregnancy: initial elevation is later followed by decrease during the end of pregnancy in rats (105). Rat placenta is comprised of decidua basalis, junctional and labyrinth zones, where PACAP and PAC1 receptor mRNAs were detected in decidual cells, as well as in chorionic vessels and stromal cells of the labyrinth zone (106). In the decidua, strongest signals were detected on day 13.5, with decreasing strength in more advanced stages. The junctional zone showed no signal, while the labyrinth zone branching villi, stem villi and chorionic vessels showed a gradually increasing signal parallel with advancing pregnancy age (106). Expression of PACAP and PAC1 receptor mRNA from human legal abortions of 6–7 weeks, from induced abortions of 14–24 weeks (second trimester) and term placentas was proven by *in situ* hybridization in stem villi and terminal villi (107). In first and second trimester samples, moderate PACAP mRNA expression was detected in stroma cells surrounding blood vessels within stem villi, while strong expression was found in full term placentas (107). Only weak expression was found in cyto- and syncytiotrophoblasts. PAC1 receptor expression showed a similar distribution pattern: stronger expression was described in the villus stroma, while weaker expression in the trophoblast cells. This increasing expression pattern of mRNA for both PACAP and its receptor suggests a potential role of the peptide in placental growth and development. Radioimmunoassay also confirmed an increase in the levels of PACAP and its specific receptor in late placentas compared to early placentas (101). Oride and colleagues reported on the presence of PACAP mRNA and PACAP immunoreactive cells in mouse primary placental cell cultures (108). PACAP expression was increased upon treatment with estradiol, progesterone, GnRH or kisspeptin. Conversely, PACAP induced kisspeptin expression in the placenta, showing that PACAP, kisspeptin, and GnRH are interrelated also at the placental level (108).

There are only a few studies dealing with the actions of PACAP in the uterus and placenta. According to a recent study, PACAP treatment leads to decrease of amplitude and an increase in frequency of myometrium contraction in pigs (103). Effects of PACAP on blood vessels and smooth muscle contractility in the uteroplacental unit was also thoroughly investigated. Preincubation with PACAP or VIP significantly inhibited the norepinephrine-induced contraction of arteries of the myometrium and stem villus in a concentration-dependent manner (100). The high concentration needed for significant relaxation indicates the necessity of local peptide release to achieve for the *in vivo* effect. Most results show that PACAP leads to placental vessel relaxation, but no effect could be observed on amplitude, tone, or frequency of strips of spontaneously contracted myometrium of pregnant women (100). Data altogether support the view that PACAP may be involved in the regulation of the uteroplacental blood flow, and results from Spencer and colleagues suggest that PACAP could facilitate endometrial blood flow, thus increasing availability of metabolic substrates to the developing decidua or the embryo (109). Involvement of PACAP placental hormone secretion has also been suggested, probably due to an induction of cAMP secretion. As PACAP and VIP acted similarly, these effects are most probably mediated by VPAC receptors (110). A recent study has detected a robust elevation of PACAP mRNA in female mice uteri with blastocyst embryos compared with non-blastocysts. Also, correlation was found between PACAP and HB-EGF (coding region of heparin-binding EGF-like growth factor) mRNA expression, which is an early embryo implantation marker. This result also supports the role of PACAP during the peri-implantation period of early mouse development (111).

Actions of PACAP have also been investigated in normal and tumorous trophoblast cells. PACAP is well-known for its general cytoprotective and survival-promoting effects in numerous cell types (19, 20). This could be confirmed in non-tumorous trophoblast cells (HTR-8/SV cells). PACAP pretreatment led to increased survival rate, increased proliferation, while it had no effect on invasion (112). However, PACAP decreased the invasion in another trophoblast cell line, HIPEC, which are invasive, proliferative extravillous trophoblast cells (112). Regulating angiogenesis may also be a function of PACAP during placental growth, as several angiogenic factors were found to be altered upon PACAP treatment of trophoblast cells (112). The disturbed intracellular signaling cascades in tumorous cells can alter the antiapoptotic, thus survival-promoting, effects of PACAP, as it has been shown in various tumour cell lines. In some tumours, PACAP has no effect on survival, while in others, PACAP is antiapoptotic, similarly to its general effects. And yet in others, PACAP is proapoptotic, thus enhances cell death, in contrast to its general protective effects. This was the case in choriocarcinoma cells, where PACAP treatment led to further decrease in survival in cells exposed to hydrogen peroxide-induced oxidative stress or chemically induced *in vitro* hypoxia (113). However, no effect was observed in lipopolysaccharide-, ethanol or methotrexate-treated cells (113, 114). Furthermore, in JAR choriocarcinoma cells, PACAP influenced the expression of several signaling molecules, such as ERK1/2, JNK, Akt, GSK, Bax, p38 MAPK (113). Altogether, these data show that PACAP and its receptors are present in the uterus and placenta, and propose some functions on blood supply, contractions, and growth both under physiological and pathological conditions, but more studies are needed to elucidate the exact function of PACAP at this level.

## Human findings

The role of PACAP in a multitude of physiological processes has drawn the attention to elucidating the physiological roles of PACAP in the human body. As the possibility of exogenous PACAP administration in humans is limited, only a few such examples are known from the literature. Regarding hormonal regulations, for example, intravenous PACAP was shown to stimulate vasopressin and PRL levels but not those of oxytocin, gonadotrophs or GH in normal men (115, 116). However, no data are available in women. Based mainly on the cytoprotective functions of PACAP, there are several promising data for its potential future therapeutic use, such as in diabetes (117), multiple sclerosis (118), the intranasal administration in neurodegenerative diseases, cognitive impairment and stroke (119–121), in form of eye drops in corneal and retinal lesions (122, 123) and dry eye disease (124). In contrast, the migraine-provoking effect of PACAP has drawn the interest towards antagonizing PACAP’s effects in migraine therapy (125, 126).

More studies are available on the distribution of PACAP in the human body and several papers have described changes of PACAP levels in different body fluids and tissues in physiological and pathological conditions. PACAP has been previously investigated in body fluids with mass spectrometry (MS), radioimmunoassay (RIA) and enzyme-linked immunosorbent assay (127), and has been found in several human body fluids: blood plasma (128–130), cerebrospinal fluid (CSF) (131) and ovarian follicular fluid (132, 133), milk (134, 135) and synovial fluid (136). The source of PACAP in human biological fluids is mainly unknown, but these studies have highlighted the potential use of PACAP as a biomarker in certain diseases, where changes can reflect the presence and/or progression of a disease (127). Among others, PACAP has a potential biomarker value in dilatative cardiomyopathy, cardiac infarct, Parkinson’s disease, migraine, polytrauma and chronic rhinosinusitis (127, 137–141). A most recent study has highlighted the potential use of PACAP, together with calcitonin gene related peptide (CGRP), in differentiating pediatric migraine from non-migraine headaches (142), while another recent study has shown the association between COVID-survival and VIP/PACAP plasma levels (143). Regarding reproductive functions, PACAP has been measured in the serum during pregnancy and delivery, and high levels of PACAP were detected in human ovarian follicular fluid, milk and amniotic fluid, as detailed below.

### PACAP in the human follicular fluid

PACAP has been detected in the human ovarian follicular fluid after superovulation treatment, with mass spectrometry (132) and radioimmunoassay (133). The potential role of PACAP in the regulation of follicular growth and maturation is further demonstrated by results showing a correlation between human follicular fluid PACAP concentration and ovarian response to superovulation treatment in infertile women (133). In this study, PACAP could be detected in all follicular fluid samples, implying an important biological role for PACAP in this culture medium for the developing oocyte. These data are in line with those demonstrating receptors for PACAP in developing follicles (92, 144). Interestingly, it appeared that low-PACAP concentrations did not correlate with the oocyte numbers: both low and high values could be measured. However, high-PACAP levels correlated with low-oocyte numbers in all cases, allowing us to conclude that below a given threshold value of PACAP it may not have a significant impact on the number of developing oocytes, while above that value, PACAP may override other intraovarian regulatory mechanisms lowering the final number of retrievable oocytes. This finding might draw attention to a derailed regulatory mechanism behind a well-known iatrogenic and potentially life-threatening condition, known as ovarian hyperstimulation syndrome (OHSS). This condition results from excessive ovarian stimulation with an incidence between 1 and 10% of IVF cycles (145). Patients have a higher chance to develop OHSS after superovulation treatment if they have significantly more follicles on the day of human chorionic gonadotropin (hCG) treatment compared with those without developing OHSS (146, 147). An earlier prospective study demonstrated that the cutoff number of developing follicles on the day of hCG administration for the occurrence of OHSS is 13 follicles (148), that is in harmony with our data (133), where a significant decrease in PACAP concentrations of the follicular fluid was found. From these a conclusion could be drawn that higher PACAP concentrations in the follicular fluid might indicate a well-regulated follicular development, while decreased concentrations could demonstrate a condition favoring the development of OHSS. The exact physiological role of PACAP in the intraovarian regulatory mechanisms influencing follicular maturation and growth is still unclear. However, based on the above data, the neuropeptide found in follicular fluid might play a role in oocyte recruitment and follicular development. Moreover, it appears that higher PACAP concentrations are associated with lower number of developing oocytes, while low PACAP concentrations might correlate with a significantly higher number of retrievable ova, thus predicting a higher chance for ovarian hyperstimulation.

### PACAP during pregnancy and in human amniotic fluid

During pregnancy, plasma PACAP38-like immunoreactivity (PACAP38-LI) was found increased in the 2nd and 3rd trimesters, indicating that the neuropeptide might be synthesized by either the placenta or other maternal tissues (33). However, in the same study, a rapid decrease in maternal plasma PACAP level could be found during labour, which might indicate a role between PACAP synthesis/function and the uteroplacental circulation and/or uterine contractions. Three days after delivery the PACAP38-LI decreased to normal levels (33). These data are not surprising, because PACAP38 was earlier detected with RIA and immunocytochemistry in each part of the uteroplacental unit (100). Further supporting the view of PACAP having significant role in placental functions, full-term placentas showed stronger PACAP38-LI on both the maternal and fetal sides, while PACAP27-LI increased only on the maternal side (101).

The amniotic fluid is a complex biological fluid, initially deriving from maternal plasma and passing through fetal membranes according to hydrostatic and osmotic pressure (149). Composition of the fluid is similar to that of fetal plasma until fetal skin keratinization, which usually occurs between 19 and 20 weeks of gestation. In a recent study, amniotic fluid samples were collected between the 15–19th weeks of gestation from volunteering pregnant women undergoing amniocentesis as a prenatal diagnostic tool. Samples were processed to detect PACAP38-LI with radioimmunoassay (150), revealing PACAP38-LI in each amniotic fluid sample, with an average level of 401 ± 142 fmol/ml. Earlier data showed higher levels of PACAP in maternal serum in late pregnancy (33) and the increasing content of PACAP in the placenta during pregnancy (101), indicating its probable placental and/or maternal origin. The higher PACAP levels found in umbilical arteries compared to the umbilical veins suggest fetal PACAP synthesis (33). Based on above results and the fact that the composition of amniotic fluid is similar to fetal plasma in this period (151), we can suggest a fetal and/or placental origin of PACAP in the amniotic fluid, with a yet unknown physiological role.

### PACAP in the human milk

Experimental data suggest that PACAP is involved in the regulation of lactation and milk ejection *via* influencing prolactin and oxytocin production and release. However, the central regulatory role of PACAP in these processes is not yet clear, as contradictory data are available on the effects of PACAP on prolactin secretion (152). While no effect was also described, stimulatory and even inhibitory effects on prolactin release have also been found depending on the route of administration, on the *in vitro* conditions and on the timing of the injections (153, 154). Prolactin mRNA was found to be stimulated by PACAP, but injection into the arcuate nucleus reduces concentration of prolactin in the plasma (51). Oxytocin has also been described to be stimulated by PACAP (155). Regarding human data, extremely high levels of PACAP-LI were measured in the human milk by RIA (134), exceeding those of plasma by about 10 times. Even higher levels were measured in the colostrum compared to transitional and mature human milk samples (135, 156). During the first 10 months of lactation, a stable high level can be observed (135). The presence of these high levels was also confirmed in domestic animals the milk of which is commonly consumed and in human milk formulas (157–159). Although the exact function of PACAP in the milk is not known at the moment, it can be suggested that it is either needed for the postnatal development or for the growth of the mammary gland itself, as several effects on the growth, differentiation and proliferation on mammary glandular epithelial cells have been described (156, 159, 160).

**Figure 1:**
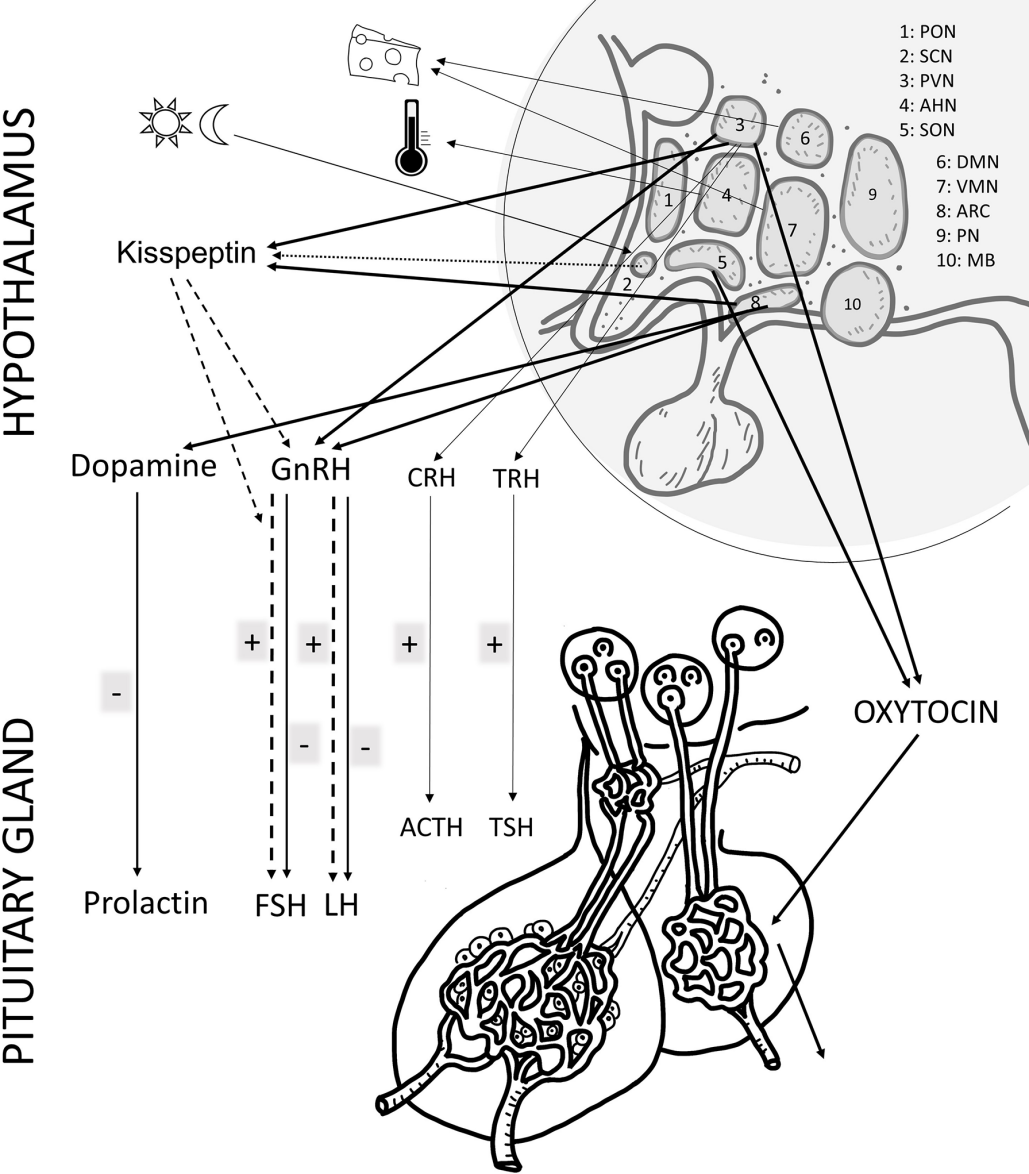
Schematic drawing of the main effects of PACAP in the female reproductive system at hypothalamic and pituitary. The main hormones/factors playing a role in reproduction and influenced by PACAP are highlighted. hypothalamic nuclei, PON (preoptic), SCH (suprachiasmatic), PVN (paraventricular), AHN (anterior hypothalamic), SON (supraoptic), DMN (dorsomedial), VMN (ventromedial), ARC (arcuate), PN (posterior), MB (mammillary body). GnRH: gonadotropin releasing hormone; TRH, thyreotropin releasing hormone; CRH, corticotropin releasing hormone; GHRH, growth hormone releasing hormone; FSH, follicule stimulating hormone; LH, luteinizing hormone; ACTH, adrenocorticotropic hormone; GH, growth hormone; ADH, antidiuretic hormone.

**Figure 2:**
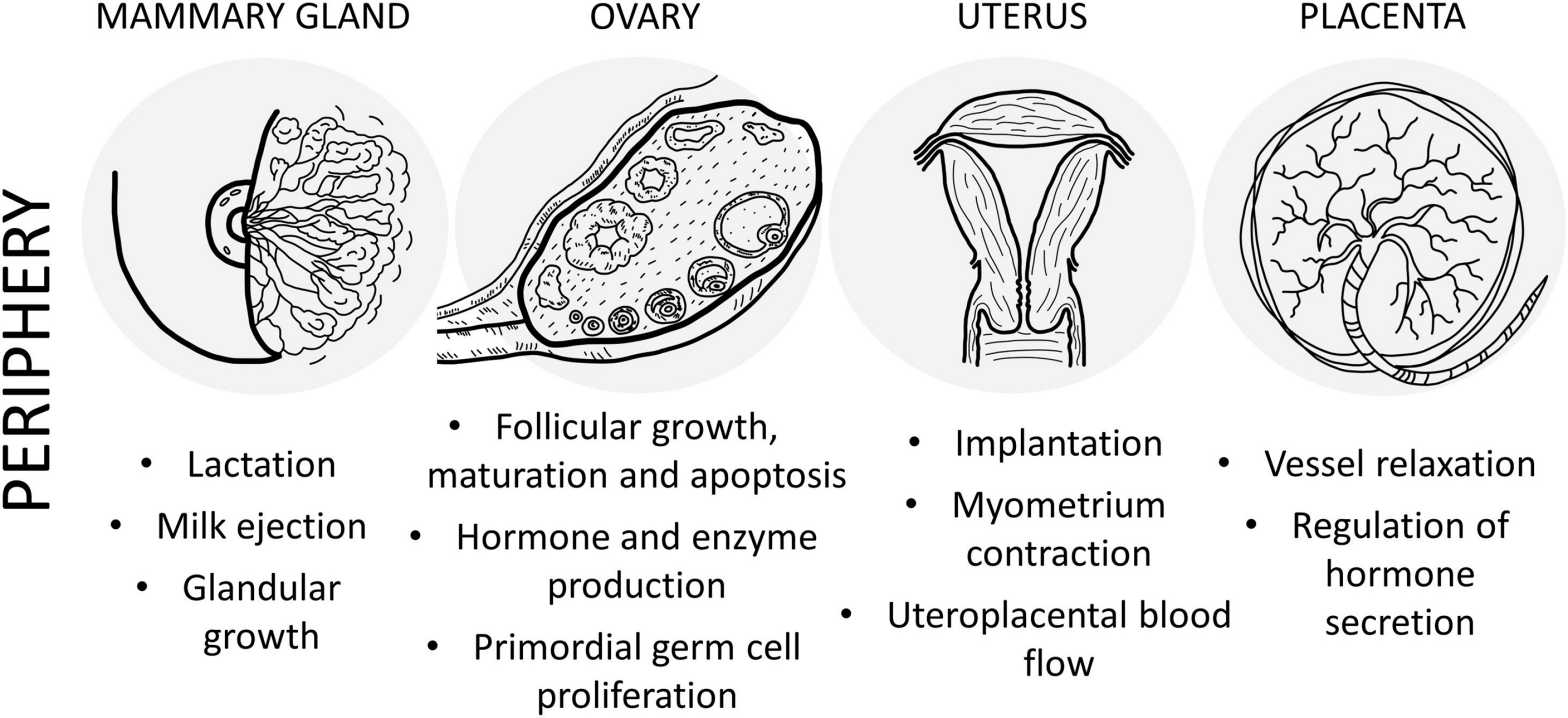
Schematic drawing of the main effects of PACAP in the female reproductive system at peripheral level.

In summary, in the present review we summarized main findings on PACAP and reproduction (**Figures 1**, **2**). As seen from the experimental and human data, PACAP and its receptors are present in the hypothalamo-hypophyseal system, in the gonads and in the uterus and placenta. Several roles of PACAP have been described in the central regulation of the reproductive functions, although there are still controversial issues that need to be resolved. In addition, the peptide influences reproductive functions in the periphery, at the ovarian and placental levels. Human data indicate that PACAP is present not only in the reproductive tissues and brain, but can also be detected in the follicular and amniotic fluid, and levels change during pregnancy. In addition, PACAP can be found in the mammary gland and milk, however, its exact function at this level still awaits future investigation. Recent data have provided evidence that PACAP might be a central regulator of puberty and female hormonal cycles, *via* interactions with the kisspeptin-GnRH system. The clinical importance of kisspeptin in several diseases has been highlighted in recent publications (161, 162). Studies summarized in the present review prove that PACAP is both a central and peripheral modulator of reproductive functions and call for further investigations to elucidate the exact role in some processes and to evaluate the potential diagnostic and/or therapeutic use of PACAP in biological fluids as a biomarker, as it has been also shown for the other players in these complex regulatory mechanisms.

## Author contributions

MK, ZN, IB, DR conceptualized, and wrote the manuscript, conceptualized and designed the figure. All authors contributed to the article and approved the submitted version.

## Funding

Supported by the Thematic Excellence Program 2021 Health Sub-program of the Ministry for Innovation and Technology in Hungary, within the framework of the EGA-16 project of the Pecs of University. This study was supported by the National Research, Development, and Innovation Fund (FK129190, K119759, K135457); National Brain Research Program (NAP2017-1.2.1-NKP-2017-00002); ELKH-TKI-14016; FIKP III. Project No. TKP2020-IKA-08 was implemented with the support provided from the National Research, Development, and Innovation Fund of Hungary, financed under the 2020-4.1.1-TKP2020 funding scheme.

## Acknowledgments

The authors thank Lili Schwieters for proofreading the manuscript.

## Conflict of interest

The authors declare that the research was conducted in the absence of any commercial or financial relationships that could be construed as a potential conflict of interest.

## Publisher’s note

All claims expressed in this article are solely those of the authors and do not necessarily represent those of their affiliated organizations, or those of the publisher, the editors and the reviewers. Any product that may be evaluated in this article, or claim that may be made by its manufacturer, is not guaranteed or endorsed by the publisher.
